# Fostering Autonomy: Exploring Innovative Dementia Care Environments in Four Countries

**DOI:** 10.1093/geroni/igaf122.1150

**Published:** 2025-12-31

**Authors:** Hilde Verbeek, Karin Wolf-Ostermann, Margaret Calkins

## Abstract

Dementia is one of the major age-related diseases world-wide and challenges not only people living with dementia and their caregivers, but also societies and health care systems as a whole. To better meet the needs of people living with dementia, innovative care environments are being developed worldwide, as part of the wider community. Aiming to strengthen independence and slow down the cognitive decline, efforts concentrate on continuously engaging people living with dementia in activities of daily life, despite a progression of the disease. This international symposium will provide four presentations on innovative dementia care environments in four different countries, which stimulate and support autonomy of older people living with dementia in an active daily life. It examines diverse environmental elements of the care environment, including organizational, social and physical aspects, and what their impact is on residents and their caregivers. The first presentation explores Green Care Farms in the Netherlands, focusing on the impact of organizational environment, in particular culture and staff’s task integration, on residents’ autonomy. The second presenter discusses results from two Dementia Village models in Canada, exploring the environmental effect on residents’ quality of life and staff’s care practices. The third presentation examines the impact of the neighborhood-built environment on social health of older residents living with dementia in Germany, using Geographical Information Systen (GIS) analyses. Finally, the last presenter describes the effects of adaptation in the physical environment, i.e. smart, ambient bright light on residents with dementia living in nursing homes in the United States.

